# Validation of an Individualized Measure of Quality of Life, Patient Generated Index, for Use with People with Parkinson's Disease

**DOI:** 10.1155/2020/6916135

**Published:** 2020-03-30

**Authors:** Ayse Kuspinar, Kedar K. V. Mate, Anne-Louise Lafontaine, Nancy Mayo

**Affiliations:** ^1^School of Rehabilitation Science, McMaster University, Hamilton, ON, Canada; ^2^Center for Outcomes Research and Evaluation, McGill University Health Centre-Research Institute, Montreal, QC, Canada; ^3^Neurology, McGill University Health Centre, Montreal, QC, Canada; ^4^Department of Neurology and Neurosurgery, McGill University, Montreal, QC, Canada; ^5^School of Physical and Occupational Therapy, McGill University, Montreal, QC, Canada

## Abstract

**Methods:**

Patients with PD completed the PGI and various standard patient-reported outcome (PRO) measures. The PGI and standard PRO measures were compared at the total score, domain, and item levels. Pearson's correlations and independent *t*-tests were used, as well as positive and negative predictive values.

**Results:**

The sample (*n* = 76) had a mean age of 69 (standard deviation 9) and were predominantly men (59%). The PGI was moderately correlated (*r* = −0.35) with the standardized disease-specific QOL measure Parkinson's Disease Questionnaire (PDQ-8). Within one severity rating, agreement between the PGI and different standard outcome measures ranged from 85 to 100% for walking, 69 to 100% for fatigue, 38 to 75% for depression, and 20 to 80% for memory/concentration.

**Conclusion:**

This study demonstrates that nominated areas of QOL on the PGI provide comparable results to standard PRO measures, and provides evidence in support of the validity of this individualized measure in PD.

## 1. Introduction

Parkinson's disease (PD) affects all aspects of an individual's life and is heterogeneous across people and time. People with PD experience a wide range of symptoms, with some of the most common being limitations in walking [1], fatigue [2], cognitive decline [3], and depression [4].

Assessing quality of life (QOL) has moved to the forefront of clinical research and practice in PD [4, 5]. The assessment of QOL is important because it can help identify the aspects of a patient's functioning that are affected by his or her disease and that are potential problems that need to be addressed during treatment. QOL assessments can help prioritize problems, set therapy goals, enhance communication between the clinician and the patient, and help monitor changes or responses to treatment [6].

Standard patient-reported outcome (PRO) measures of QOL generally have predetermined domains and response options (e.g., PDQ-39, SF-36). The underlying assumption with these measures is that (i) the same domains of life are important to all people, and (ii) for each domain, all people have the same needs and goals [7]. Furthermore, these measures assume that there is equal weighting between the domains, or if weights do exist, they are based on values obtained from the general population who have never experienced the disease [8].

Individualized measures of QOL allow patients to identify the domains (or areas of life) that are important to them and to assign a weight on the relative importance of each domain. The Patient Generated Index (PGI) is an individualized measure of QOL (IQOL) that allows patients to nominate, rate, and value areas that have the most impact on their quality of life. The validity of the PGI has been tested in other health conditions [9–12], but not yet in PD. Therefore, the objective of this study is to estimate how well areas of QOL identified on the PGI (an individualized measure) agree with ratings obtained from standard PRO measures in people with PD.

## 2. Materials and Methods

### 2.1. Subjects

Patients with PD were recruited from the McGill Movement Disorder Clinic in Montreal, Canada. Patients were excluded if they presented with a major comorbid condition (e.g., severe dementia, severe psychiatric, neurological, or other medical condition likely to have major impact on the quality of life, other than PD). During the routine clinical visit, patients were introduced to the study and those interested were presented with a consent form. The protocol was approved by the local Institutional Ethics Review Board.

### 2.2. Measures

#### 2.2.1. Individualized Measure of Quality of Life

*(1) Patient Generated Index*. The Patient Generated Index (PGI) is a short individualized measure of QOL that takes only a couple of minutes to administer [11, 13]. First, participants are asked to identify up to five of the most important areas of their lives affected by PD. Second, they are asked to rate the extent to which they are in the selected areas on a scale of 0 to 10, where 0 is the worst they can imagine and 10 exactly as they would like to be. In the third phase, patients are given twelve spending “points” to allocate across the areas. The more points a patient spends for an area, the more important that area is. The PGI is calculated as the (sum of (area score × points spent/12)) [14]. The PGI produces a total score of overall QOL from 0 to 10, where higher scores indicate better QOL. The PGI has been shown to be reliable, valid, and responsive to change [15].

#### 2.2.2. Standard Patient-Reported Outcome Measures

*(1) Parkinson's Disease Questionnaire-8*. The Parkinson's Disease Questionnaire-8 (PDQ-8) [16] is a self-reported measure of QOL that includes questions on mobility, activities of daily living, emotional well-being, stigma, social support, cognition, communication, and bodily discomfort. Each question is scored from 0 to 4 points and the scores are summed. The summed score is then divided by the total possible score and reported as a percentage out of 100, where lower scores indicate better quality of life. The PDQ-8 has demonstrated moderate correlations with standard measures of disease severity [16].

*(2) RAND-36 Physical Function Index*. The Physical Function Index (PFI) of the RAND-36 [17] is composed of 10 questions that describe a range of physical activities including limitations with walking. Each item has 3 response options “yes, limited a lot,” “yes, limited a little,” and “no, not limited at all.” The sum of the responses is taken and scaled from 0 to 100.

*(3) Geriatric Depression Scale*. The Geriatric Depression Scale (GDS) [18] is an 8-item self-report measure designed to identify depression in the elderly. Examples of questions in the GDS include “are you basically satisfied with your life?” and “do you feel that your life is empty?”. Response options for the 8 items are “yes/no,” and one point is assigned to each answer. The measure is scored from 0 to 8, where higher scores indicate more depression.

*(4) Apathy Scale*. The Apathy Scale (AS) [19] is an interviewer-administered questionnaire designed to measure loss of motivation, interest, and social engagement. It consists of 14 questions with response options “not at all,” “slightly,” “some,” or “a lot.” Scores range from 0 to 42, and higher scores indicate more severe apathy. The AS has demonstrated reliability and validity in PD [19].

*(5) Perceived Deficits Questionnaire-20*. The Perceived Deficits Questionnaire-20 (PDQ-20) [20] is a self-reported measure of perceived cognitive function. It contains 20 items that assess various domains of cognition: attention, memory, planning, and organization. Response options are in a 5-point Likert scale, from “never” to “almost always” and rates of “0” to “4” are given according to the response option selected. The total score is out of 80, where higher scores indicate greater cognitive impairment. The PDQ-20 has demonstrated good reliability and validity [20].

*(6) Visual Analogue Scale (VAS) for Fatigue*. Patients were presented with a visual analogue scale from 0 (much fatigue) to 10 (no fatigue) and were asked to mark a line on the scale to indicate how much fatigue they were experiencing. The VAS for fatigue has demonstrated acceptable convergent validity against self-reported measures of fatigue [21].

*(7) Visual Analogue Scale (VAS) for Depression*. Patients were presented with a visual analogue scale from 0 (much depression) to 10 (no depression) and were asked to mark a line on the scale to indicate what they were experiencing. The VAS for depression has been shown to be significantly correlated with a standardized and lengthier measure of depression (the Beck Depression Inventory) [22].

### 2.3. Statistical Methods

The PGI and the standard PRO measures were compared at the (1) total score level, (2) domain level, and (3) item level. A domain refers to broad HRQL concepts (e.g., depression), whereas an item refers to a specific question in a PRO measure.

#### 2.3.1. Total Score Level

At the total score level, Pearson's correlation and coefficient of determination were calculated to estimate the strength of the association between the PGI and the PDQ-8 (a standard PRO measure of QOL in PD). We hypothesized that the correlation coefficient value between the PGI and the PDQ-8 would be moderate (0.3 to 0.4) [23].

#### 2.3.2. Domain Level

At the domain level, the independent *t*-test or the Mann–Whitney *U* test was calculated, as appropriate, to compare total scores from standard PRO measures that assessed walking (RAND-36 PFI), depression (GDS), and memory/concentration (PDQ-20), between people who nominated and did not nominate those corresponding areas using the PGI. The Shapiro–Wilk test was used to verify the assumption of normality. If the condition of normality was not met, the Mann–Whitney *U* test was used. We hypothesized that scores on the standard PROs would be statistically significantly different between individuals who nominated and did not nominate a domain.

#### 2.3.3. Item Level

All items on the standard PRO measures were transformed to range from 0 to 10 (0 as the worst level and 10 as the best level) to enable direct comparison between them. As the standardized PRO measures had varying numbers of response options (3, 4, or 5), the PGI severity score (on a scale from 0 to 10) was adapted to match these response categories (see Table 1).

Previous studies have demonstrated that, on a scale of 0–10 (where 0 is none and 10 is severe), a score of 4 or greater for either pain or fatigue is considered a concerning level and requires further comprehensive assessment [24–26]. Thus, since the PGI has a reversed scale (where 0 is severe and 10 is none), the threshold for a concerning score was set at 6. In other words, a severity score from 0 to 6 on the PGI was defined as concerning, and a score from 7 to 10 was defined as nonconcerning.

At the item level, agreement and positive predictive values (PPVs) and negative predictive values (NPVs) were calculated for walking, fatigue, depression, and memory/concentration. To estimate PPV and NPV, the score on the standard PRO item represented the true value, and the PGI nominated area was the value to be assessed. The PPV was computed as the number of patients who responded in the concerning range on the standard PRO measure (0 to 6) divided by the total number of patients who nominated that domain on the PGI. A severity score from 0 to 6 out of 10 would be considered concerning, so response categories on standardized PRO measures that fell within this range were also defined as concerning (as shown in Table 1). The NPV was calculated as the number of patients who responded in the nonconcerning range on the standard PRO measure (7 to 10) divided by the number of patients who did not nominate that domain on the PGI.

## 3. Results

### 3.1. Sample Characteristics

Table 2 presents a summary of the sample characteristics. Participants had an average age of 69 (standard deviation: 9.5) and 59% were men. The average number of years since diagnosis was 6, and the average number of years since symptom onset was 8. Participants had an average of 2.3 and median of 2.0 on the Hoehn and Yahr scale. The mean total score for the PGI was 4.2 (standard deviation: 1.8) on a scale from 0 to 10, where higher scores indicate better quality of life. The average total score for the PDQ-8 was 26.99 (standard deviation: 14.2).

### 3.2. PGI Responses

**Figure 1:**
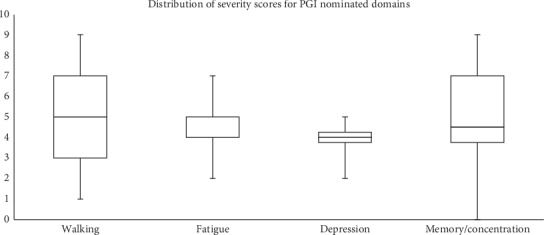
Distribution of the severity scores on a scale from 0 (worst) to 10 (best) for walking, fatigue, depression, and memory/concentration among people who endorsed the area. 0 is the worst they can imagine and 10 exactly as they would like to be.

The domains generated from the PGI were categorized using the World Health Organization's International Classification of Functioning, Disability, and Health (ICF). The ICF provided a coding framework and common nomenclature for each nominated area. The results of this process have been published previously [27]. The domains reported by patients on the PGI included dexterity, walking, sleep, fatigue, cognition, tremors, sports, depression, anxiety, self-care, speech, socializing, household tasks, bowel and bladder, work/employment, and balance.  Figure 1 presents the distribution of the severity rating scores (scaled from 0 to 10) for the PGI domains: walking, depression, and memory/concentration. The borders of the box are the upper (75%) and lower (25%) quartiles, and the horizontal line inside the box is the median. The median severity rating scores for walking, fatigue, depression, and memory/concentration were 5, 4, 4, and 4.5, respectively.

### 3.3. Validity Testing

#### 3.3.1. Total Score Level

Pearson's correlation coefficient between the PGI total score and the PDQ-8, a standard PRO measure of QOL, was *r* = −0.35 (*r*^2^ = 0.12; *p*=0.006). The distribution of the PDQ-8 and the PGI is provided in the Supplementary Materials (Figures [Supplementary-material supplementary-material-1] and [Supplementary-material supplementary-material-1]).

#### 3.3.2. Domain Level

Table 3 presents the results of an independent *t*-test or Mann–Whitney *U* test for participants who nominated walking, depression, and memory/concentration on the PGI, in comparison with participants who did not select these areas. The independent *t*-test was used for the PDQ-20. The Mann–Whitney *U* test was used for the RAND-36 PFI and the GDS because the assumptions of normality were not met. Participants who nominated walking, depression, and memory/concentration on the PGI had significantly worse scores on the related standard PRO measure than participants who did not nominate these domains. For example, the median score on the RAND-36 PFI was significantly lower (*p*=0.0006) for participants who nominated walking as an area of concern (40.0) compared to participants who did not nominate walking (80.0). Similar results were observed on the GDS for depression and the PDQ-20 for cognition.

#### 3.3.3. Item Level

Table 4 presents the extent to which severity ratings on the PGI agreed with the severity rating on related standard PRO measures. The table shows the results for the percent agreement within one severity level, and the PPV and NPV for detecting a concerning level of walking, fatigue, depression, or memory/concentration on standard PRO measures.

There were three items related to walking from the RAND-36 PFI (walking one block, walking several blocks, and walking more than a kilometer). Percent agreement within one severity level ranged from 85% to 100% for these items. If walking was nominated on the PGI, the PPV for a concerning score across the three standard PRO items in the RAND-36 PFI ranged from 55% to 80%; if walking was not nominated, the NPV ranged from 59% to 80%.

For fatigue, the PGI severity rating was compared against one item from the AS (energy for daily activities) and one item from a fatigue-specific VAS (much fatigue to no fatigue). Percent agreement between the PGI severity rating and the standard PRO items was 69% (energy for daily activities) and 100% (VAS—much fatigue to no fatigue). The PPV for a concerning score across standard PROs was between 69% and 94% when fatigue was nominated, and the NPV was lower at 33% and 41% when fatigue was not nominated.

For depression, the PGI was compared against one item that was a depression-specific VAS (much depressed to no depression) and one item from the PDQ-8 (felt depressed). Percent agreement within one severity level was 38% (PDQ-8—felt depressed) and 75% (VAS—much depression to no depression). The PPV was between 85% and 86%, and the NPV was between 63% and 66%.

For memory/concentration, the PGI was compared against 20 items from the PDQ-20. Percent agreement between the PGI and the PDQ-20 items ranged from 20% to 80%. Seven out of 20 items demonstrated ≥70% agreement between the PGI and the standard PRO. Eleven out of 20 items had percent agreement values between 50% and 69%. Only two items had percent agreement values less than 50%. PPV values ranged from 27% to 93% and the NPV values ranged from 37% to 92%. Table 4 presents the results for the cognition items that demonstrated the highest percent agreement (≥70%).

## 4. Discussion

This study evaluated the construct validity of an individualized measure of QOL, the PGI, in people with PD. The results provided support for the validity of the scores produced by the PGI in comparison with standard PRO measures.

Patients who nominated walking, fatigue, depression, and memory/concentration as an area of concern (regardless of whether it was rated poorly or not) scored significantly lower on corresponding standard PRO measures, thus supporting construct validity of the PGI. Furthermore, agreement between PGI severity ratings and corresponding standard PRO items were relatively high for walking and fatigue. For depression, there was high agreement with the VAS but not with the depression item from the PDQ-8 (how often during the last month have you felt depressed? Never, Occasionally, Sometimes, Often, and Always). The VAS scale might have performed better because it has a 10-point scale similar to the PGI. For cognition, the PGI demonstrated moderate to high agreement with most of the items from the PDQ-20, with the exception of two. These two items were in relation to planning and organization, which were not nominated as an area of concern by our sample.

The PGI was moderately correlated with the PDQ-8, a standard QOL measure developed for people with PD. These results were in line with our hypothesis, as previous research has demonstrated moderate associations between IQOL measures and standard measures of QOL (e.g., subscales of the SF-36) [28]. Strong correlations are typically not observed because the domains nominated by patients can be different from what are included in standard measures developed by researchers. Although convergent validity may be lower in IQOL measures, content validity, on the contrary, is quite high. Content validity is the degree to which the content of an instrument is an adequate reflection of the construct to be measured [29]. IQOL measures have high content validity because the individual determines what constitutes his/her QOL [30]. Individualization of items maximizes content validity and guarantees their importance to every participant in a study.

The PGI domains nominated by patients in our sample were diverse and included dexterity, walking, sleep, fatigue, cognition, tremors, sports, depression, anxiety, self-care, speech, socializing, household tasks, bowel and bladder, work/employment, and balance. Although some of these domains are included in the PDQ-8, several are not. This may explain the moderate correlation observed between the two measures. We may have observed larger correlation values if the PGI had been compared with the more comprehensive version of the PDQ-8, the PDQ-39. Marinus and colleagues reported in a systematic review that the content between different PD-specific HRQL measures differed considerably [31]. The authors reported that although the PDQ-39 included most domains important to people with PD, it lacked items on self-image, nighttime sleep problems, sexual activity, and transfers. Furthermore, our research team recently assessed the content validity of generic preference-based measures of HRQL (i.e., utility measures) such as the EQ-5D in PD [27]. These HRQL measures are typically used to evaluate the cost-effectiveness of different interventions. Our results showed that several domains important to people with PD were missing in these generic measures and that the development of a disease-specific preference-based measure may be warranted for use in cost-effectiveness analysis in PD.

The magnitude of the correlation is also affected by the range of the variables understudy [32]. For example, putting the PGI, PFI, and PDQ-8, all on the same scale (0–100 with 100 best) and calculating confidence intervals, even the upper bound of the CI for the PGI (mean: 42; 95% CI: 40–44) is well outside of the lower bound of the other measures (PDQ-8 transformed mean: 73; 95% CI: 71–75; PFI mean: 64; 95% CI: 60–67). Thus, there are more people at the lower end of the PGI than there are in the other 2 measures. In addition, the PGI usually captures only areas of concern whereas with other measures, the value is driven by not having deficits that are queried. These factors have been reported before [10], and thus, strong correlations are not expected.

For at least one item per PGI area, the PPV for the PGI was high. In other words, nominating an area was highly predictive of responding equivalently on a standardized item (see Table 4). For example, nominating walking as an area affecting quality of life was 80% predictive of being unable to walk more than a kilometer, and nominating fatigue (or depression) was 94% (86%) predictive of rating fatigue (depression) as high. NPV values were not as high as expected because just because an area is not nominated does not mean the person does not have the problem, it is just not as important as those nominated.

Scores observed on the various PRO measures in our sample were similar to the literature. Jenkinson and Fitzpatrick reported on the PDQ-8 in 121 people with PD in Canada [33]. The average PDQ-8 score for their sample was 31.4, which was similar to the average score of 27.0 in our sample. Both samples were similar in disease severity and duration. In addition, individuals who nominated walking as an area of concern on the PGI had similar PFI values to previous studies that have used this measure in PD [34–36]. People who did not nominate walking as an area of concern had a mean of 72/100, which is similar to published normative data for the same age group (mean 75.7/100) [37]. For the PDQ-20, individuals who nominated memory or concentration as an area of concern had a mean value of 35/80, which is close to the cutoff value of ≥40 that is indicative of cognitive impairment [38]. Individuals who did not nominate this area as a concern had a considerably lower mean value of 22/80 on the PDQ-20.

One of the advantages of IQOL measures is that they use a patient-centered approach. IQOL measures capture aspects of QOL that are most important to the individual. Unlike standard PRO measures, IQOL does not consist of predetermined domains. They allow the individual to nominate the domains that are important to the quality of his/her life. What further distinguishes IQOL from standard measures is that they allow the individual to determine the relative importance of each domain [8]. Standard measures assume that there is equal weighting between the domains measured. Another advantage of IQOL measures that is not commonly reported is their ability to serve as powerful evaluative instruments [8]. An evaluative measure is one that is responsive to changes in individuals or groups over time. Individualization of questionnaires can maximize responsiveness because (1) the domains are nominated by the patient (high content validity); (2) consequently, it facilitates the detection of the “signal” or true change; and (3) it minimizes and may eliminate floor and ceiling effects [30]. This study was cross-sectional, but future research should evaluate the responsiveness of the PGI in people with PD.

IQOL measures also have the potential to be used in clinical practice [8]. Standard PRO measures of QOL have often been difficult to use in clinical practice because the results are not easily interpretable by clinicians [39]. Clinicians at times face an uncertainty as to what the scores mean and how to apply the information [39]. On the other hand, IQOL measures may be simpler to use and interpret than standard PRO measures. The PGI can allow clinicians to quickly identify the areas that are of concern to the patient and that need to be addressed during treatment. Furthermore, scoring each domain from 0 to 10 allows them to assess the impact of the disease on each domain, and the distribution of the “points” among the domains determines the relative importance of each. A further advantage of IQOL instruments is that they can improve communication between the patient and the physician [40]. For rehabilitation professionals, IQOL measures can help prioritize treatment options by identifying the areas that the patient wants to improve the most. The domains generated by patients can be allowed to vary over time or they can be kept the same over time. In a clinical setting, clinicians may prefer to show patients the domains that were identified at baseline again at the follow-up visit to monitor how their patient's HRQL changes over time. All in all, clinicians need measures that are patient-centered, easy to use, clinically relevant, and sensitive to change—criteria that may be met by IQOL measures.

A limitation of this study is the small sample size and that patients with PD were recruited from only one clinical site. Further research should be conducted on a larger sample size and longitudinal validity (i.e., responsiveness) of the PGI in PD should be tested.

## 5. Conclusions

In conclusion, the PGI is an IQOL that allows patients to nominate, rate, and value areas that have the most impact on their quality of life. This study demonstrated that nominated areas of QOL on the PGI provided comparable information to standard PRO measures, and presented evidence in support of the validity of personalized measures in PD.

## Figures and Tables

**Table 1:** Categorization of response options on standard PRO measures and PGI severity rating.

Standard patient-reported outcome measures	Patient Generated Index
3 categories	0–3; 4–6; 7–10
4 categories	0–3; 4–6; 7–8; 9–10
5 categories	0–1; 2–3; 4–6; 7–8; 9–10

0 is the worst they can imagine and 10 exactly as they would like to be.

**Table 2:** Demographic and clinical characteristics of the sample (*n* = 76).

Characteristics	Mean (SD) or *N* (%)
Age (y)	69.1 (9.5)
Women	31 (41%)
Years since diagnosis	6.0 (4.1)
Years since symptom onset	8.2 (5.1)
Tremor dominant/akinetic rigid	46 (71)/19 (29)
History of falls (none/rare/monthly)	41 (63)/16 (25)/8 (12)
Levodopa equivalent dose (mg/day)	770.4 (523.7)
Hoehn and Yahr stage (1–5)	2.3 (0.9)
Patient Generated Index (10–0)	4.2 (1.8)
Parkinson's Disease Questionnaire-8 (0–100)	27 (14.2)
RAND-36 Physical Function Index (0–100)^*∗*^	63.9 (27.7)
Geriatric Depression Scale (0–8)^*∗∗*^	2.1 (2.4)
Perceived Deficits Questionnaire-20 (0–80)^*∗∗∗*^	25.8 (11.9)
Apathy Scale (0–42)^*∗∗∗∗*^	22.3 (3.9)

SD, standard deviation; *N*, number. ^*∗*^*N* = 64; ^*∗∗*^*N* = 70; ^*∗∗∗*^*N* = 67; ^*∗∗∗∗*^*N* = 66. Patient Generated Index: 0 is the worst QOL, and 10 is perfect QOL. Hoehn and Yahr Stages: 1—minimal or no functional disability; 2—symptoms, no impairment of balance; 3—mild to moderate disability, still physically independent; 4—severe disability but still able to walk and stand unassisted; 5—confined to bed or wheelchair unless aided. Parkinson's Disease Questionnaire-8: lower is better; RAND-36 Physical Function Index: higher is better; Geriatric Depression Scale: higher is more depression; Perceived Deficits Questionnaire-20: higher is more deficits, ≥40 considered cognitively impaired; Apathy Scale: high is more apathy.

**Table 3:** Comparison of the PGI against standard PRO measures at the domain level among people who endorsed the area.

	Identified as an area of concern on the PGI Mean (SD) Median (*Q*1–*Q*3) (*N*)	Not identified as an area of concern on the PGI Mean (SD) Median (*Q*1–*Q*3) (*N*)	Independent *t*-test or Mann–Whitney *U* test, *p* value
Walking (higher is better)	45.0 (27.3)	71.9 (24.0)	0.0006
RAND-36 physical function index	40.0 (25.0–65.0) (19)	80.0 (50.0–95.0) (46)	

Depression (higher is worse)	4.5 (2.8)	1.8 (2.2)	0.009
Geriatric depression scale	4.5 (2.5–7.0) (8)	1.0 (0.0–3.0) (62)	

Memory/concentration (higher is worse)	34.6 (12.1)	23.4 (10.6)	0.0004
Perceived Deficits Questionnaire-20	36.5 (25.0–44.0) (15)	22.0 (17.0–30.0) (52)	

SD, standard deviation; *N*, number; *Q*1, quartile 1; *Q*3, quartile 3.

**Table 4:** Comparison of the PGI against standard PRO measures at the item level among people who endorsed the area.

Items	Number of response options	Standard PRO measure	Agreement Δ ± 1 *N* (%)	PPV (%)	NPV (%)
Walking					
Walking several blocks	3	RAND-36 PFI	20 (100)	70	63
Walking one block	3	RAND-36 PFI	18 (90)	55	80
Walking more than a kilometer	3	RAND-36 PFI	17 (85)	80	59

Fatigue					
Energy for daily activities	4	AS	16 (100)	69	41
Much fatigue to none	10	VAS	11 (69)	94	33

Depression					
Much depression to no depression	10	VAS	6 (75)	86	63
Felt depressed	5	PDQ-8	3 (38)	85	66

Memory/concentration					
Forget if you had already done something	5	PDQ-20	12 (80)	80	75
Forget what you came into the room for	5	PDQ-20	11 (73)	67	46
Trouble concentrating	5	PDQ-20	11 (73)	53	70
Forget what you did the night before	5	PDQ-20	11 (73)	53	70
Find your mind drifting	5	PDQ-20	11 (73)	67	56
Forget what you did last weekend	5	PDQ-20	11 (73)	67	62
Forget to take your medication	5	PDQ-20	11 (73)	67	74

*N*, number; PPV, positive predictive value; NPV, negative predictive value; PFI, Physical Function Index; AS, Apathy Scale; VAS, visual analogue scale; PDQ-20, Perceived Deficits Questionnaire-20.

## Data Availability

The data used to support the findings of this study are restricted by the McGill University Health Center Research Ethics Board in order to protect patient privacy. Data are available from Nancy Mayo (nancy.mayo@mcgill.ca) for researchers who meet the criteria for access to confidential data.

## Supplementary Materials

Supplementary Materials Figure 2: distribution of the PDQ-8. Figure 3: distribution of the PGI.
