# Supplementary figures and images for: Validation of an Individualized Measure of Quality of Life, Patient Generated Index, for Use with People with Parkinson's Disease
Source: Neurol Res Int. 2020 Mar 30;2020:6916135. doi: 10.1155/2020/6916135 (PMC7149443; doi:10.1155/2020/6916135)

**APPENDIX**

**Figure 2.** Distribution of the PDQ-8


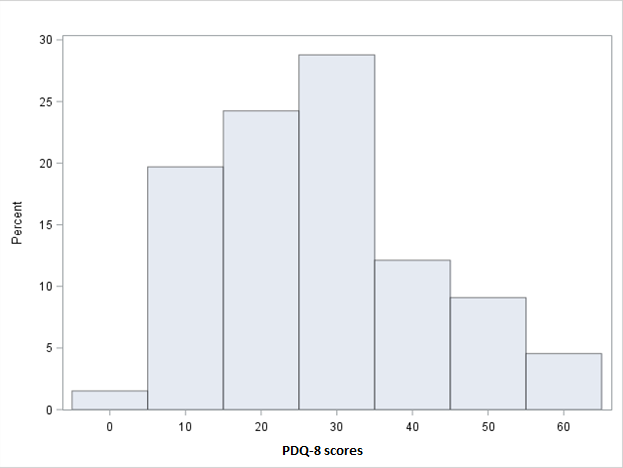


**Figure 3.** Distribution of the PGI


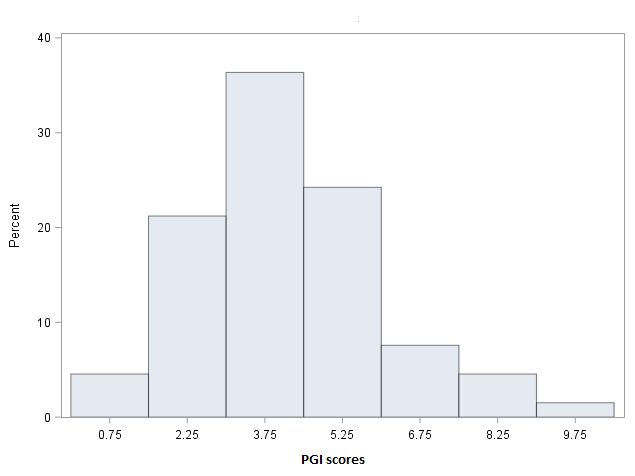

Supplement: Supplementary Materials — Figure 2: distribution of the PDQ-8. Figure 3: distribution of the PGI. [file 6916135.f1.docx]
